# Small fish, large river: Surprisingly minimal genetic structure in a dispersal‐limited, habitat specialist fish

**DOI:** 10.1002/ece3.6064

**Published:** 2020-02-06

**Authors:** Brooke A. Washburn, Mollie F. Cashner, Rebecca E. Blanton

**Affiliations:** ^1^ Department of Biology Center of Excellence for Field Biology Austin Peay State University Clarksville TN USA; ^2^ Present address: Department of Biological Sciences University of Denver Denver CO USA

**Keywords:** dispersal‐limited, isolation‐by‐distance, linear river system, patchy habitat, small spatial scale, stepping‐stone dispersal

## Abstract

Genetic connectivity is expected to be lower in species with limited dispersal ability and a high degree of habitat specialization (intrinsic factors). Also, gene flow is predicted to be limited by habitat conditions such as physical barriers and geographic distance (extrinsic factors). We investigated the effects of distance, intervening pools, and rapids on gene flow in a species, the Tuxedo Darter (*Etheostoma lemniscatum*), a habitat specialist that is presumed to be dispersal‐limited. We predicted that the interplay between these intrinsic and extrinsic factors would limit dispersal and lead to genetic structure even at the small spatial scale of the species range (a 38.6 km river reach). The simple linear distribution of *E. lemniscatum* allowed for an ideal test of how these factors acted on gene flow and allowed us to test expectations (e.g., isolation‐by‐distance) of linearly distributed species. Using 20 microsatellites from 163 individuals collected from 18 habitat patches, we observed low levels of genetic structure that were related to geographic distance and rapids, though these factors were not barriers to gene flow. Pools separating habitat patches did not contribute to any observed genetic structure. Overall, *E. lemniscatum* maintains gene flow across its range and is comprised of a single population. Due to the linear distribution of the species, a stepping‐stone model of dispersal best explains the maintenance of gene flow across its small range. In general, our observation of higher‐than‐expected connectivity likely stems from an adaptation to disperse due to temporally unstable and patchy habitat.

## Graphical abstract

We examine whether physical obstacles and geographic distance influence genetic structure in a presumably dispersal‐limited, habitat specialist riverine fish. We found minimal levels of genetic structure associated with rapids and distance. So, we conclude that there are minimal filters to gene flow in this endangered species and that higher‐than‐expected connectivity likely stems from an adaptation to disperse due to temporally unstable and patchy habitats.

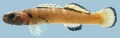

## 1 INTRODUCTION

Understanding dispersal and gene flow is fundamental to many ecology, evolution, and conservation biology studies, as both contribute to population persistence through space and time. Intrinsic species traits (e.g., dispersal ability, habitat specialization) and extrinsic habitat conditions (e.g., physical barriers, geographic distance) often dictate the amount of population connectivity and gene flow in a species. Organisms with moderate to high dispersal have higher recolonization potential (Albanese, Angermeier, & Peterson, 2009; Hanski & Simberloff, 1997) and less commonly exhibit reduced genetic diversity stemming from genetic drift and inbreeding, making them less vulnerable to extinction (Frankham, 1995; Whiteley, Fitzpatrick, Funk, & Tallmon, 2015). Species with limited dispersal ability and high habitat specificity typically have greater genetic structure and more risk of depleted genetic diversity (Pilger, Gido, Propst, Whitney, & Turner, 2017; Savage, Fremier, & Shaffer, 2010; Sterling, Reed, Noonan, & Warren, 2012) than those with high dispersal ability and generalist habitat requirements (Canal, Roques, Negro, & Sarasola, 2017; Reid, Wilson, Carl, & Zorn, 2008; Row et al., 2012). This trend is dictated primarily by the patchy distribution of specialist habitats. Habitat patchiness makes dispersal risky, while continuous generalist habitats facilitate more successful dispersal (Gottelli, Sillero‐Zubiri, Marino, Funk, & Wang, 2013; Turner, 2001; Wagner & McCune, 2009).

However, many studies show unexpected levels of genetic structure that seem to contradict these patterns. For instance, high levels of genetic structure at small spatial scales in *Pyrrhura orcesi* (El Oro parakeet) and *Salmo trutta* (Brown trout) were found to be due to unsuitable habitat in dispersal corridors overriding their presumed inherent high dispersal ability (Klauke, Schaefer, Bauer, & Segelbacher, 2016; Stelkens, Jaffuel, Escher, & Wedekind, 2012). Conversely, hypothesized fine‐scale genetic structure due to supposed low dispersal abilities was not found in *Ammocrypta pellucida* (Eastern Sand Darter) and *Xenicus gilviventris* (New Zealand Rock Wren) (Ginson, Walter, Mandrak, Beneteau, & Heath, 2015; Weston, Taylor, & Robertson, 2016). Deviations from expectations of genetic structure based on dispersal ability or habitat specialization are frequently attributed to anthropogenic habitat fragmentation, which can override high dispersal ability and lead to decreased population connectivity and higher‐than‐expected levels of genetic structure (Janecka et al., 2016; Richmond, Backlin, Galst‐Cavalcante, O'Brien, & Fisher, 2018; Roberts, Angermeier, & Hallerman, 2013). Alternatively, some habitat specialists exhibit evidence of high gene flow (Ginson et al., 2015; Peled, Shanas, Granjon, & Ben‐Shlomo, 2016), as dispersal may be an adaptation to isolated, patchy, and unstable habitats (Centeno‐Cuadros, Román, Delibes, & Godoy, 2011; Peterson & Denno, 1998).

The influence of extrinsic habitat features and intrinsic species traits on dispersal and gene flow may be more pronounced in riverine systems than terrestrial environments because dispersal pathways among optimal habitat patches in rivers are more limited. Habitat patches are typically arranged linearly or in a dendritic pattern since intervening terrestrial habitats are inhospitable for aquatic organisms, restricting the potential routes for movement among patches (Fagan, 2002; Hughes, Schmidt, & Finn, 2009). As a result, barriers to dispersal are more likely to limit gene flow, leading to more severe impacts on population and species viability in lotic environments (Ward, Woodwark, & Skibinski, 1994). The unique structure of riverine systems also leads to the expectation of a downstream increase in genetic diversity (DIGD; Paz‐Vinas, Loot, Stevens, & Blanchet, 2015). Downstream increase in genetic diversity is attributed to unidirectional stream flow (upstream to downstream) that may cause asymmetric downstream dispersal, leading to loss of genetic diversity in upstream areas (Morrissey & de Kerckhove, 2009; Paz‐Vinas et al., 2015; Thomaz, Christie, & Knowles, 2016). This downstream‐biased dispersal can also lead to greater genetic differentiation in upstream populations compared to downstream populations (Finn, Bonada, Múrria, & Hughes, 2011; Paz‐Vinas & Blanchet, 2015).

Few studies have examined the impacts of patchy habitat on dispersal and gene flow in obligate riverine species. Of these, only a small subset have examined genetic effects on small‐bodied, benthic fishes, which include some of the most imperiled fishes in North America (Jelks et al., 2008). Most existing studies have focused on the hierarchical genetic structure and diversity within river systems or drainages with dendritic population arrangements (Austin, Jelks, Tate, Johnson, & Jordan, 2011; Roberts et al., 2013; Robinson, Simmons, Williams, & Moyer, 2013); however, less is known about levels of structure and diversity within a single, linear portion of a river. In this study, we examine a fish species found only in the mainstem of the Big South Fork River (Cumberland River drainage). Since the species does not live in any of the river's tributaries, there is no dendritic riverine structure to influence gene flow, making this study one of the few to examine genetic diversity and structure in a linear system. This simple linear system minimizes options for dispersal, allowing for better characterization of the impacts of intrinsic species traits on spatial genetic structure (Kanno, Vokoun, & Letcher, 2011) and easier interpretation of how extrinsic habitat factors contribute to dispersal and gene flow. Additionally, genetic structure should not be influenced by any anthropogenic physical barriers to dispersal since the portion of the river the species occupies contains no channel modifications (e.g., channelization, dredging) or human‐made in‐stream barriers such as dams or weirs.

**Figure 1:**
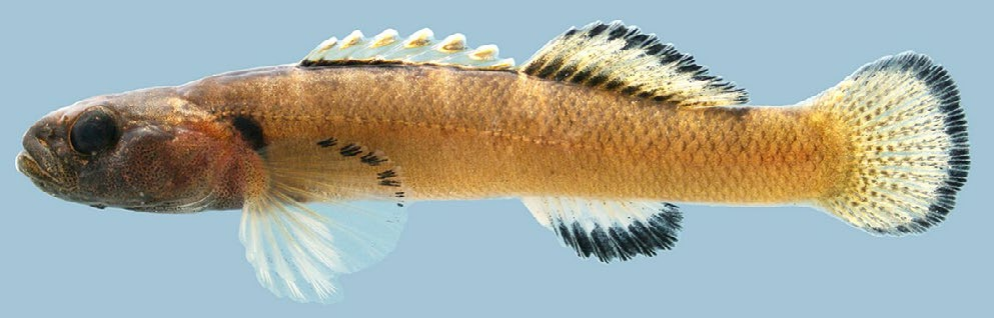
A male Tuxedo Darter (*Etheostoma lemniscatum*) in breeding coloration (photograph by Matthew R. Thomas)

Our focal taxon is the federally endangered Tuxedo Darter, *Etheostoma lemniscatum* (Figure 1). This endemic fish is found in only a 38.6 km reach of the mainstem Big South Fork River where its upstream range is constrained by a Class IV rapid, Angel Falls, that is thought to impede upstream movement (Davis & Cook, 2010), and its downstream range is limited due to inundation effects (e.g., sedimentation and reduced flow) from a reservoir created by Wolf Creek Dam in 1950 (Campbell, Risk, Andrews, Palmer‐Bell, & MacGregor, 1990). This species is expected to show high levels of genetic structure, even at small spatial scales, since it is a habitat specialist with hypothesized limited dispersal ability (Centeno‐Cuadros et al., 2011; Fluker, Kuhajda, & Harris, 2014). It has several features associated with limited dispersal ability: it is a small‐bodied, benthic darter (Knouft & Page, 2003) that lacks a swim bladder (Page, 1983), has high reproductive investment (Turner & Trexler, 1998), demersal eggs laid on the undersides of rocks (Eisenhour & Burr, 2000), and benthic larvae (Douglas et al., 2013; Wallus & Simon, 2005). *Etheostoma lemniscatum* is a habitat specialist of shallow, slow‐moving water with cobble or slab‐rock substrate, generally located adjacent to and upstream of riffles, and is typically found at depths less than 1 m (Davis & Cook, 2010; Eisenhour & Burr, 2000). Less than ~25% of the river is this depth or shallower (McConkey, 2010); thus, this specialist species may occupy less than 25% of the total area available in its environment. It utilizes this habitat year round for both feeding and spawning, so dispersal into different breeding habitats is not expected (Davis & Cook, 2010; Eisenhour & Burr, 2000).

There are also several extrinsic habitat features of the river system that may further shape genetic structure in *E. lemniscatum*. Its specific microhabitat is patchily distributed and separated by deep pools that can be over 1 km long and up to 15 m deep (Davis & Cook, 2010; Eisenhour & Burr, 2000; McConkey, 2010). These pools may limit movement among patches, as no *E. lemniscatum* have been observed in these habitats (Davis & Cook, 2010; Eisenhour & Burr, 2000). Also, there is an 11.7 km stream reach where few historical localities exist, and the presence of *E. lemniscatum* is rare (Blanton & Jenkins, 2008; Davis & Cook, 2010; Eisenhour & Burr, 2000). This reach coincides with an area where the Big South Fork narrows significantly, changing the geomorphology and creating a long series of rapids where little to no optimal habitat for *E. lemniscatum* occurs (Eisenhour & Burr, 2000; Phillips et al., 2010). Tributaries in this reach have historically experienced heavy coal mining, which also may have degraded habitat quality and constrained the reach's viability as a dispersal corridor (Eisenhour & Burr, 2000; Rikard, Kunkle, & Wilson, 1986). This reach terminates in a high gradient Class IV rapid, Devil's Jump, that may function as a dispersal barrier in the same manner that Angel Falls is believed to prevent dispersal at the upstream end of the species range (Davis & Cook, 2010). These features of the Big South Fork, particularly the 11.7 km stream reach containing little suitable habitat and Devil's Jump (hereon referred to as Devil's Jump Disjunction [DJD]), may act as significant barriers or filters to *E. lemniscatum* dispersal and, thus, gene flow.

Because this linear study system mirrors the arrangement of Kimura and Weiss's (1964) one‐dimensional stepping‐stone model, our first objective was to determine whether gene flow in this system follows this classic model and leads to isolation‐by‐distance, especially since this is expected of an organism with low dispersal ability and a linear distribution (Slatkin, 1993; Wright, 1943). Second, we examined whether the intervening pools, distance, or rapids act as filters or barriers to gene flow among habitat patches, as predicted for a habitat specialist, dispersal‐limited species. A third objective was to examine whether our system shows the expected patterns of the distribution of genetic diversity in riverine systems (DIGD and increased upstream genetic differentiation). We also provide a baseline understanding of genetic diversity in *E. lemniscatum* to help guide future conservation decisions, given that the species displays several intrinsic characteristics (habitat specialist, dispersal‐limited, benthic larvae, and small native range) indicative of an elevated extinction risk and vulnerability to anthropogenic habitat fragmentation (Douglas et al., 2013; Warren et al., 2000).

## 2 METHODS

### 2.1 Sample collection

**Figure 2:**
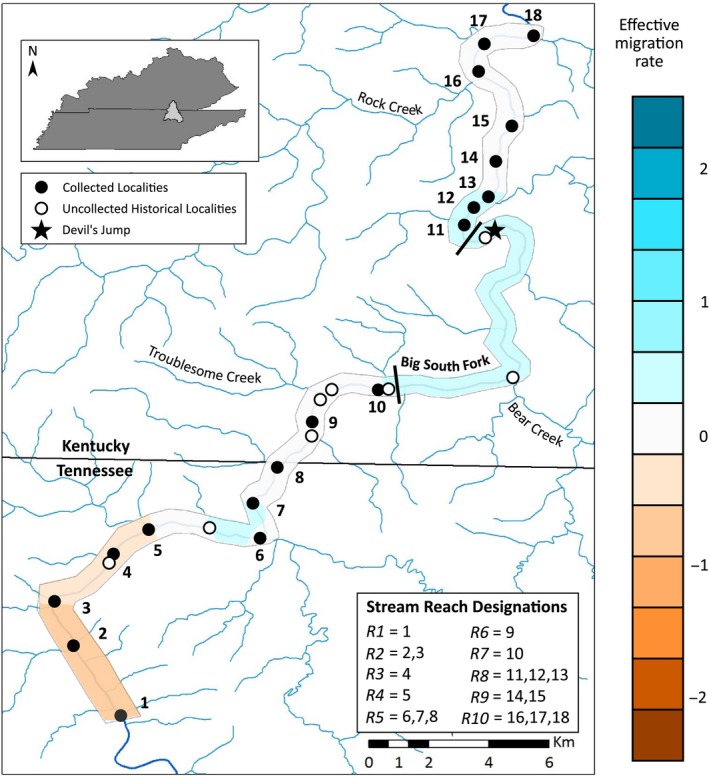
Known localities for *Etheostoma lemniscatum* in the Big South Fork River across Kentucky and Tennessee. Locality numbers by black circles correspond to Table S2. Locality 1 is the upstream‐most sample site. Reach groupings (*R1*–*R10*) are designated in bottom right legend and correspond to reaches used in Table 1. River section between black bars represents Devil's jump disjunction (DJD) where little optimal habitat for *E. lemniscatum* occurs. Overlay on river is estimated effective migration surfaces plot from EEMS analysis showing deviations from an exact isolation‐by‐distance (IBD) model. Areas in blue have higher effective migration rates than expected (corridors for gene flow), areas in orange have lower effective migration rates than expected (barriers to gene flow), and areas in white are those consistent with IBD

In August–October 2015, we sampled *E. lemniscatum* from 18 of the 26 known localities, encompassing its range (Figure 2). We surveyed a nineteenth site near the mouth of Bear Creek but found no *E. lemniscatum*. Individuals of *E. lemniscatum* were collected while snorkeling, using a hand‐held dipnet. We took fin clips from the upper portion of the caudal fin and preserved in 95% ethanol from all captured individuals. Fish recovered and, subsequently, were released at the capture site. We recorded geographic coordinates at each surveyed habitat patch.

### 2.2 Microsatellite genotyping

We extracted whole genomic DNA from fin clips using either a Thermo Fisher Scientific GeneJet Genomic DNA Purification Kit or a Qiagen DNeasy Blood and Tissue Kit. Hereditec (Lansing, NY, USA) developed species‐specific microsatellite primers, which identified over 6,500 potential loci for this study. Of these, we optimized 92 loci for *E. lemniscatum* using gradient reactions to identify the optimal annealing temperature for successful amplification of a given locus. Sixty‐eight loci successfully amplified for *E. lemniscatum*, but only 20 were polymorphic and easily scored, and subsequently used in five multiplex reactions (see Table S1 for variability of these loci in *E. lemniscatum*). PCRs were 10 μl total volume. For samples above 5 ng/μl DNA concentration, the PCR contained 5.65 μl PCR pure water, 1.00 μl 10X concentrate standard Taq reaction buffer‐Mg free (New England Biolabs, Inc.), 1.20 μl 25 mM MgCl_2_ (New England Biolabs, Inc.), 0.20 μl 10 mM dNTPs (New England Biolabs, Inc.), 0.25 μl 10 p.m. forward primer, 0.50 μl 10 p.m. reverse primer, 0.10 μl 5,000 U/ml Taq Polymerase (New England Biolabs, Inc.), 0.10 μl M13‐labeled primer (Applied Biosystems, Inc.), and 1.00 μl DNA. Samples with DNA concentrations below 5 ng/μl differed in containing 4.65 μl PCR pure water, and 2.00 μl DNA, with all other reagent amounts the same as those with higher DNA concentrations. PCR cycle conditions were as follows: initial denaturation at 94°C for 1‐min; 35 cycles each of denaturation at 94°C for 30‐s, primer specific annealing temperature ranging from 60°C–65°C (see Table S1) for 30‐s, extension at 72°C for 30‐s; and a final extension at 72°C for 5‐min.

PCR products were genotyped using an ABI3730 sequencer with LIZ600 as the size standard at the University of Florida Interdisciplinary Center for Biotechnology Research. Allele sizes were scored automatically using the panel editor function in GeneMarker v1.6 (SoftGenetics LLC) and then confirmed manually. Any allele that did not fit the expected repeat pattern for that locus was closely examined and then, if necessary, edited manually or reamplified and regenotyped. To ensure consistency of allele sizes across reactions, three positive controls were included on each genotyping run.

### 2.3 Marker validation and genetic diversity

To meet sample size requirements for some analyses used, sites where *n* ≤ 6 individuals were grouped together with the closest adjacent site(s) to create 10 stream reaches (Figure 2). To examine genetic structure across DJD, reaches upstream (*R1*–*R7*) and downstream (*R8*–*R10*) of DJD were grouped prior to analyses.

Microsatellite loci were evaluated for evidence of scoring errors due to null alleles, large allele dropout, and stutter with MICRO‐CHECKER v2.2.3 (Van Oosterhout, Hutchinson, Wills, & Shipley, 2004) using 1,000 simulations with a 95% confidence interval (CI). Departures from Hardy–Weinberg equilibrium (HWE) per locus and per reach were tested using exact tests. Linkage disequilibrium was tested across all locus pairs to ensure independence of loci. Both HWE and linkage disequilibrium were evaluated using Markov chain parameters (10,000 dememorization steps, 1,000 batches, 10,000 iterations per batch) in GENEPOP v4.4 (Rousset, 2008) and, since multiple tests were done, the subsequent *p*‐values had a sequential Bonferroni correction applied to minimize type‐I errors (Rice, 1989). The following measures of genetic diversity were determined for the 10 reaches, and the species overall: mean number of alleles per locus (*N*_a_), and observed (*H*_o_) and expected heterozygosity (*H*_e_) in GenAlEx v6.503 (Peakall & Smouse, 2012); allelic richness (AR) and private allelic richness (PAR), metrics that use rarefaction to account for uneven sample sizes, in HP‐RARE v1.1 (Kalinowski, 2005); and inbreeding coefficient (*F*_IS_) in GENETIX v4.05 (Belkhir, Porsa, Chikhi, Raufaste, & Bonhomme, 2004), using 10,000 permutations to assess significance. To assess whether there was DIGD from asymmetric downstream dispersal, we calculated the distance between our most‐upstream reach and all reaches downstream, then ran a linear regression on three of our measures of genetic diversity (AR, PAR, and *H*_o_) at each reach against the calculated distances in R v3.4.2 (R Core Team., 2017).

We estimated *N*_e_ for the entire species using the linkage disequilibrium (LD) method in NeESTIMATOR v2.01 (Do et al., 2014; Waples & Do, 2008), which estimates *N*_e_ from single‐year datasets. The upper and lower bounds of *N*_e_ were determined via the parametric 95% CI option (Waples, 2006). Since the inclusion of rare alleles can upwardly bias *N*_e_ with the LD method, we excluded alleles with a frequency of <0.02 to provide a more conservative estimate (Waples & Do, 2010).

We tested for recent population declines in our 10 reaches, and the species overall with the program BOTTLENECK v1.2.02 (Cornuet & Luikart, 1996; Piry, Luikart, & Cornuet, 1999). The two‐phase model with 0%, 10%, and 20% multistep mutations, and 36% variance was tested for significance via a Wilcoxon sign‐rank test (Di Rienzo et al., 1994; Peery et al., 2012). Various multistep mutation percentages were examined in our tests following recommendations of Peery et al. (2012).

### 2.4 Spatial genetic structure

The extent of genetic differentiation between the 10 reaches and sites upstream and downstream of DJD was first evaluated with pairwise *F*_ST_ values in GENETIX v4.05 (Belkhir et al., 2004), which were assessed for significance with 10,000 permutations. We then used pairwise *F*_ST_ values and pairwise log‐transformed riverine distances (determined in ARCGIS v10.2.2 [ESRI]) for our 10 reaches to test for isolation‐by‐distance (IBD) using IBD v1.52 (Bohonak, 2002). The pairwise riverine distances between our 10 reaches account for distances between, but not within, reaches (i.e., the distance between downstream‐most site of a reach to the upstream‐most site of the subsequent reach; Table 2). We tested for a significant relationship between geographic distance and genetic distance using a Mantel test with 10,000 randomizations.

We examined population substructure using the program STRUCTURE v2.3.4 (Pritchard, Stephens, & Donnelly, 2000). We used the following parameters: no a priori population affiliation assumed, a genetic admixture model, allele frequencies correlated, 5 iterations for each value of *K* examined (*K* = 1–20), and 10,000 burn‐in Markov chain Monte Carlo (MCMC) steps followed by 100,000 MCMC steps. Since our initial STRUCTURE runs failed to detect any population structure, subsequent STRUCTURE analyses employed the LOCPRIOR model using our 10 reaches and reaches upstream and downstream of DJD as priors. Hubisz, Falush, Stephens, and Pritchard (2009) suggest using the LOCPRIOR model when genetic divergence is low and difficult to detect. We then assessed the most likely number of population clusters using the mean log‐likelihood (Ln[Pr(X|*K*)]) (Pritchard et al., 2000) and Δ*K* (Evanno, Regnaut, & Goudet, 2005) methods in the program STRUCTURE HARVESTER web v0.6.94 (Earl & VonHoldt, 2012). When there was evidence for more than one cluster, we ran hierarchical structure analysis (each indicated cluster was subsequently examined for hidden structure) as suggested by Janes et al. (2017). Additionally, when multiple clusters were suggested, the output for multiple independent runs of each *K* was summarized and visually represented using the main pipeline in the program CLUMPAK (Kopelman, Mayzel, Jakobsson, Rosenberg, & Mayrose, 2015). As another means to visualize grouping of individuals from our 10 stream reaches, we conducted a principal components analysis (PCA). The PCA was performed in R v3.6.1 (R Core Team, 2019) using ADEGENET v2.1.1 package (Jombart, 2008) via the *dudi.pca* function from the ADE4 v1.7‐13 package (Dray & Dufour, 2007). As recommended by Jombart (2008), missing data were replaced with site‐specific mean allele frequencies.

To examine fine‐scale gene flow and further assess the effect of riverine distance on genetic structure in our study system, we conducted a spatial autocorrelation analysis in GenAlEx v6.503. For geographic distances, we used riverine distances calculated via ARCGIS v10.2.2 (ESRI) between our original 18 sites. A significant and positive genetic autocorrelation coefficient (*r*) for individuals within a specified distance class is an indication of deviation (*r* ≠ 0) from the null hypothesis of no spatial genetic pattern (*r* = 0) (Peakall, Ruibal, & Lindenmayer, 2003). Therefore, when multiple increasing distance classes are tested, the limit at which nonrandom (positive) spatial autocorrelation of individual genotypes (or genetic patch size) ends can be detected from the first x‐intercept in the correlogram, provided a significant *r* is present in at least one distance class (Peakall et al., 2003; Smouse & Peakall, 1999; Weston et al., 2016). We ran the analysis with several different distance class breakdowns to ensure we had approximately equal and sufficient sample sizes per distance class and to check that distance classes did not exceed the scale of genetic structure in *E. lemniscatum* (Peakall et al., 2003). In our first analysis, we examined spatial autocorrelation with 11 distance classes spanning the 38.6 km range of the species. To verify that within‐site spatial autocorrelation was not driving the significant positive autocorrelation of the 1 km distance class in the first analysis, we ran a second analysis with 8 distance classes spanning 0–7.5 km (all individuals of the same site fell within the 0 km distance class). Significance of our spatial autocorrelation analyses was assessed with 9,999 bootstraps which calculated 95% error bars around each distance class estimate of *r*, and 9,999 permutations that calculated the 95% CI of the null hypothesis of *r* = 0. Using a conservative approach, an estimate of *r* for an individual distance class was only considered significant when *r* both exceeded the CI of the null hypothesis of zero and when the 95% error bars for *r* did not cross the *x*‐axis of *r* = 0 (Peakall et al., 2003).

To visualize areas in our study system that may be corridors or barriers to gene flow, we ran the program EEMS (estimated effective migration surfaces; Petkova, Novembre, & Stephens, 2016). This method was selected because it is best implemented in systems expected to exhibit broad signals of IBD (like our linear river system) and because it is more robust to uneven sampling schemes than other analyses, such as principal components analysis and STRUCTURE (Petkova et al., 2016; Puechmaille, 2016). Estimated effective migration surfaces identifies areas where gene flow deviates from exact IBD using a stepping‐stone model (Kimura & Weiss, 1964) to calculate deviations from IBD between adjacent demes: Areas with higher effective migration rates than expected are corridors to gene flow, while areas with lower effective migration rates are barriers. During preliminary runs of EEMS, we adjusted our parameters to attain the recommended 20%–30% acceptance rates which helps with MCMC convergence when the analysis is run (Petkova et al., 2016). To confirm that grid size did not influence our results, we ran the analysis using different starting seeds across three grid sizes (100, 500, and 900 demes) with 10,000,000 MCMC steps sampled every 5,000 steps, after an initial burn‐in of 1,000,000 steps. We then assessed MCMC convergence across the individual runs using log‐posterior plots. Finally, we combined results from across the three grid sizes into a final plot using the REEMSPLOTS R package (Petkova et al., 2016).

## 3 RESULTS

### 3.1 Sampling, marker validation, and genetic diversity

We observed 271 individuals of *E. lemniscatum* from 18 sites (Figure 2; Table S2). Of these, we captured 163 individuals for genetic analyses. The average number of individuals observed per site was 15; sites 2, 4, 5, 9, 10, and 14 had the highest abundances (Table S2). All 163 individuals were successfully genotyped at 16 or more loci (23/3260 total genotypes were missing; <1% missing). Since no evidence of scoring errors (from stutter, allele dropout, or null alleles), no departures from HWE, and no consistent evidence of linkage disequilibrium among locus pairs were observed, all 20 loci were included in further analyses.

A total of 120 different alleles were amplified across all samples. Across all loci, the average was 6.0 alleles/locus (range: 2–12 alleles). Measures of allelic diversity were low overall, but fairly uniform among our ten reaches; *H*_o_ and *H*_e_ were also relatively similar across reaches (Table 1). All reaches had private alleles, but PAR was relatively higher in *R9* (PAR = 0.25) compared to other stream reaches (PAR range: 0.05–0.16; Table 1). All three genetic diversity metrics in our linear regressions (AR, PAR, and *H*_o_) showed no significant downstream increase in genetic diversity (all: *R* ^2^ < .30, *p* > .10). Most reaches had *F*_IS_ values that did not significantly differ from zero; however, *R3* and *R9* had significant negative *F*_IS_ values, indicating outbreeding occurring in those reaches (Table 1). Our multiple tests for bottlenecks with different parameters were congruent and showed evidence of a bottleneck in *R2*, *R3*, *R4*, *R6*, *R8*, *R10*, and in the species overall (Table 1). No reach displayed evidence for deviation from HWE after Bonferroni correction (Table 1). The *N*_e_ estimate for the species was low; the mean value was 497 individuals, with a 95% CI of 315–1,060 individuals.

**Table 1:** Measures of genetic diversity for each of the 10 stream reaches and for *Etheostoma lemniscatum* overall: mean alleles per locus (*N*_a_), allelic richness (AR), private allelic richness (PAR), observed (*H*_o_) and expected (*H*_e_) heterozygosity, inbreeding coefficient (*F*_IS_), *p*‐values for Bottleneck, and *p*‐values for Hardy–Weinberg equilibrium (HWE)

Stream reach	*n*	*N*_a_	AR	PAR	*H*_o_	*H*_e_	*F*_IS_ (95% CI)	Bottleneck	HWE
*R1*	16	4.00	3.75	0.05	0.587	0.561	−0.014 (−0.124 to 0.026)	0.053	0.780
*R2*	25	4.55	3.92	0.08	0.605	0.600	0.012 (−0.051 to 0.032)	**0.027**	0.014
*R3*	17	4.15	3.81	0.05	0.619	0.573	**−0.049 (−0.132 to −0.032)**	**0.005**	0.187
*R4*	17	4.05	3.73	0.06	0.598	0.558	−0.040 (−0.166 to 0.014)	**0.013**	0.999
*R5*	13	4.05	3.83	0.16	0.528	0.547	0.075 (−0.074 to 0.115)	0.273	0.573
*R6*	13	3.95	3.78	0.06	0.585	0.568	0.011 (−0.099 to 0.021)	**0.024**	0.191
*R7*	16	4.20	3.79	0.14	0.590	0.569	−0.004 (−0.147 to 0.070)	0.077	0.940
*R8*	13	3.90	3.77	0.12	0.603	0.571	−0.014 (−0.178 to 0.055)	**0.016**	0.492
*R9*	18	4.50	4.01	0.25	0.654	0.582	**−0.095 (−0.187 to −0.073)**	0.082	0.785
*R10*	15	3.95	3.74	0.08	0.612	0.585	−0.011 (−0.111 to 0.009)	**0.012**	0.015
Species Overall	163	6	–	–	0.601	0.597	−0.003 (−0.028 to 0.017)	**0.004**	0.196

Note

Values in bold indicate significance (based on 95% confidence intervals for *F*_IS_, *p* < .05 for bottleneck, and *p* < .002 for HWE after Bonferroni correction); *n* is the number examined in each reach. Stream reach identifiers are defined in Figure 2.

### 3.2 Spatial genetic structure

**Figure 3:**
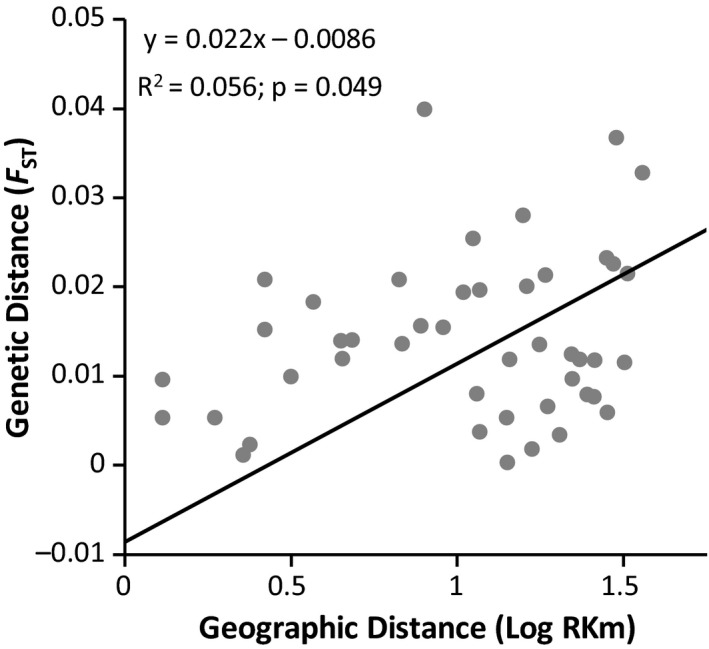
Relationship between geographic (log‐transformed river kilometers) and genetic distance (*F*_ST_) resulting from the isolation‐by‐distance (IBD) analysis of the ten stream reaches examined for *Etheostoma lemniscatum*. For corresponding *F*_ST_ values and pairwise distances between stream reaches, refer to Table 2

We observed low differentiation between the ten reaches, with pairwise *F*_ST_ values ranging from 0.0002 to 0.040 (Table 2). Of the 45 pairwise comparisons, 22 were significantly different from zero at *p* < .05; however, after Bonferroni correction, only three of the pairwise comparisons were significant (*p* < .011; Table 2). All three of these included our most‐upstream site, *R1*, and contained the highest *F*_ST_ values (*F*_ST_ = 0.033–0.040) recovered. The results of our Mantel test in the program IBD recovered a significant IBD relationship among our ten stream reaches (*R* ^2^ = .056; *p* = .049), but distance explained only 5.6% of the total genetic variation, suggesting that distance plays only a small role in creating genetic structure in *E. lemniscatum* (Figure 3). The pairwise *F*_ST_ between the reach groupings upstream and downstream of DJD was significant but low, suggesting that Devil's Jump and the 11.7 km disjunction are weak filters, but not barriers, to gene flow (*F*_ST_ = 0.004; *p* = .019).

**Table 2:** Pairwise *F*_ST_ values (above the diagonal) and pairwise distances (below the diagonal) for the 10 stream reaches examined for *Etheostoma lemniscatum* and used in our IBD analysis (Figure 3)

	*R1*	*R2*	*R3*	*R4*	*R5*	*R6*	*R7*	*R8*	*R9*	*R10*
*R1*	–	0.021*	0.021*	0.040**	0.025*	0.028*	0.021*	0.037**	0.021*	0.033**
*R2*	2.65	–	0.002	0.018*	0.014*	0.008	0.005	0.008	0.006	0.011*
*R3*	6.70	2.39	–	0.010	0.014	0.015*	0.020*	0.012	0.012*	0.023*
*R4*	8.00	3.69	1.30	–	0.010	0.016*	0.019*	0.012	0.008	0.023*
*R5*	11.17	6.86	4.47	3.17	–	0.005	0.012	0.020*	0.007	0.010
*R6*	15.78	11.47	9.08	7.78	1.87	–	0.015*	0.012	0.002	0.003
*R7*	18.43	14.12	11.73	10.43	4.52	2.65	–	0.004	0.000	0.014*
*R8*	30.17	25.86	23.47	22.17	16.26	14.39	11.74	–	0.005	0.014
*R9*	32.62	28.31	25.92	24.62	18.71	16.84	14.19	1.30	–	0.001
*R10*	36.17	31.86	29.47	28.17	22.26	20.39	17.74	4.85	2.28	–

Note

*F*_ST_ values with one asterisk (*) are significant at *p* < .05, and *F*_ST_ values in bold with two asterisks (**) are significant following Bonferroni correction (*p* < .0011). Stream reach numbers correspond to those used in Figure 2 and are listed in order from the upstream‐most (*R1*) to downstream‐most (*R10*).

**Figure 4:**
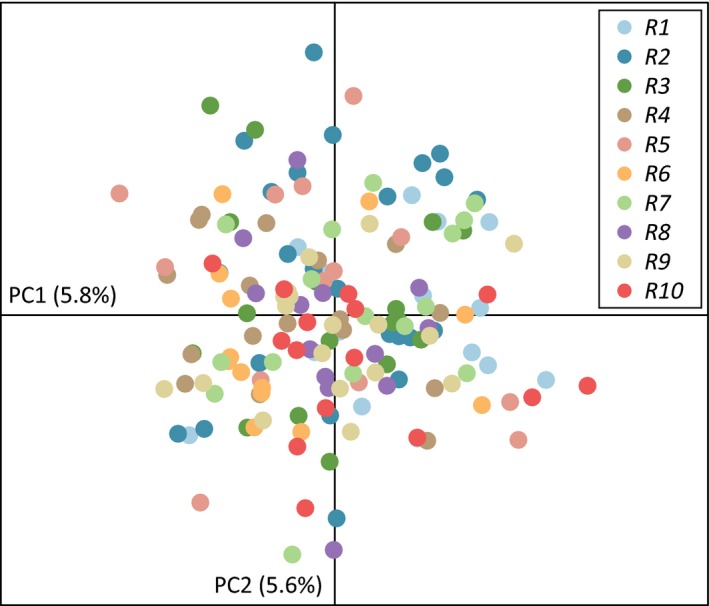
Principal components analysis (PCA) of all 163 *Etheostoma lemniscatum* individuals from all 10 reaches. Each circle is an individual and the color of the circle indicates the reach, which are denoted in the legend. The first two principal components explain 11.4% of the genetic variance (the proportion explained by each axis is included in parentheses)

The STRUCTURE run using our 10 stream reaches as a prior for the LOCPRIOR model found *K* = 1 as the most likely number of clusters using the mean log‐likelihood method (Figure S1a). The Δ*K* method indicated *K* > 1 (Figure S1a), but this method cannot detect *K* = 1 (Evanno et al., 2005). Also, the STRUCTURE plots for *K* > 1 assigned all individuals roughly equally to each suggested cluster (Figures S1b and S1c); given these factors, we disregarded *K* > 1 as a viable alternative hypothesis of population structure in *E. lemniscatum*. Our PCA results were consistent with our STRUCTURE results in recovering no population genetic structure; the 10 stream reaches showed considerable overlap in genetic variation (Figure 4). In the STRUCTURE run that used the areas upstream and downstream of DJD as the prior for the LOCPRIOR model, support for *K* = 1 or *K* = 2 was equivocal using the mean log‐likelihood method; the Δ*K* method recovered *K* = 2 (Figure S2a). The STRUCTURE plot for *K* = 2 shows evidence of admixture throughout the species range, supporting *K* = 1 as the best explanation of genetic structure in *E. lemniscatum* (Figure S2b). However, because there was some support for *K* = 2 for areas upstream and downstream of DJD, we examined those areas independently using a separate STRUCTURE run; no evidence of hidden structure was detected (Figure S3). Ultimately, all STRUCTURE analyses and our PCA indicate that *E. lemniscatum* is composed of one population.

**Figure 5:**
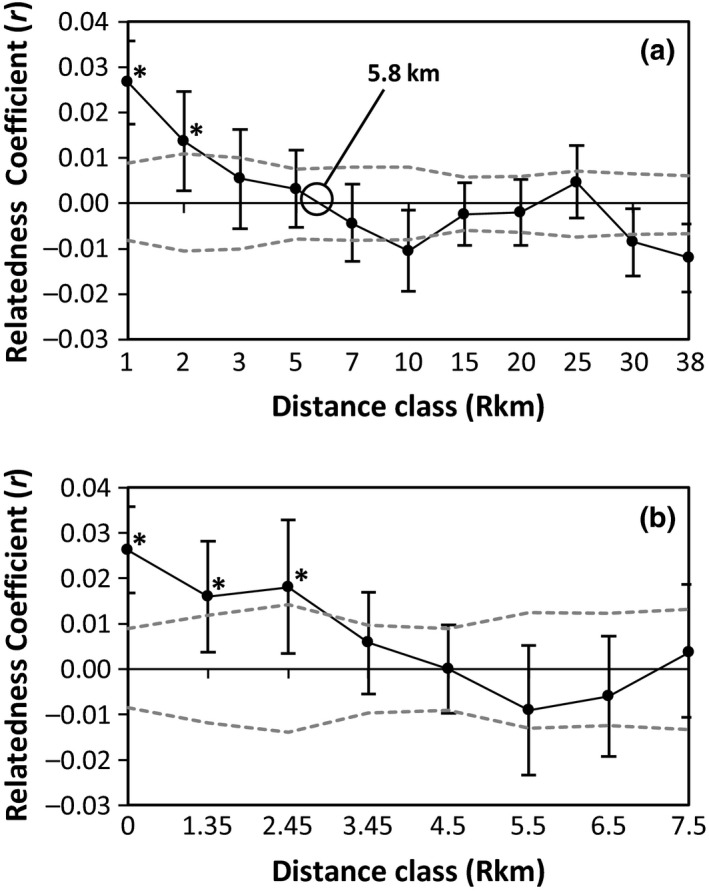
Spatial autocorrelation correlogram of the genetic autocorrelation coefficient (*r*) for (a) distance classes spanning the entire *Etheostoma lemniscatum* range and (b) distance classes from 0–7.5 km where all individuals of the same site fall within the 0 km distance class. For both, the line at *r* = 0 represents the null hypothesis of no spatial autocorrelation of individual genotypes (meaning that individual genotypes are randomly distributed across the landscape). Black circles are mean *r* values for each distance class; associated bars are 95% bootstrap errors. The dashed gray lines indicate the 95% confidence interval of the null hypothesis and were determined by permutation. Asterisks show significantly positive *r* values (*p* < .05). When a significant *r* is found in at least one distance class, the furthest extent of detectable positive spatial autocorrelation (or genetic patch size) is inferred from the first x‐intercept

Our spatial autocorrelation analysis spanning the entire range of *E. lemniscatum* detected significant positive spatial autocorrelation of individual genotypes (*p* < .01) at the first two distance classes (Figure 5a), indicating that individuals separated by 1–2 kilometers are more genetically similar than expected at random. Since we found significant positive autocorrelation, we interpreted the x‐intercept of 5.8 km (Figure 5a) as the genetic patch size. This indicates that individuals separated by more than 5.8 km are less genetically similar than individuals assigned randomly to distance classes. A second spatial autocorrelation analysis at the finer spatial scale of 0–7.5 km found significant positive spatial autocorrelation (*p* < .01) at the 0, 1.35, and 2.45 km distance classes (Figure 5b). This second analysis indicates that autocorrelation found at the 1 km distance class in the previous analysis (Figure 5a) was not driven by within‐site autocorrelation of genotypes and that we can detect significant positive spatial autocorrelation up to 2.45 km in *E. lemniscatum*.

Our EEMS analysis detected areas of relatively higher and lower rates of gene flow (Figure 2). The stretch of river where DJD occurs exhibited a slightly elevated effective migration rate from exact IBD, indicating that DJD may be a corridor for gene flow. Another area around sites 6 and 7 shows the same trend. The only area to exhibit reduced effective migration rates was the upstream area of the species range, encompassing the five most‐upstream sites, indicative of reduced gene flow to the upstream area of the species range. While all EEMS runs converged on these same results, all posterior probabilities were <0.90, meaning that none of these deviations from exact IBD were statistically supported. This indicates that effective migration, or gene flow, is functionally uniform throughout the study area, further supporting the recovery of only one population for *E. lemniscatum*.

## 4 DISCUSSION

Habitat specialists, especially those that are also dispersal‐limited, often display signatures of high genetic structure (Pilger et al., 2017; Waters & Burridge, 2016). In such species, factors such as poor habitat matrix conditions, geographic distance, and habitat fragmentation have relatively strong impacts on gene flow (Barr et al., 2015; Savage et al., 2010), often leading to isolation or reduced connectivity, even at small spatial scales (Polato et al., 2017; Storfer, Mech, Reudink, & Lew, 2014). We examined the impacts of distance, intervening pool habitats, and a disjunction in our focal taxon's range on gene flow and genetic diversity. We expected *E. lemniscatum* would exhibit reduced gene flow as predicted of a habitat specialist, dispersal‐limited species, especially given that its microhabitat is separated by long reaches of unsuitable habitat. Although we observed low levels of genetic structure (related to distance and DJD), these abiotic factors were not barriers to gene flow. We found that gene flow occurs at high levels up to 2.45 km and is well maintained up to 5.8 km, distances greater than the pool lengths separating most adjacent habitat patches. This suggests that most pools between localities do not constrain dispersal. Additionally, we found little evidence for a bias in downstream dispersal since there was no significant trend of DIGD and only minimal signals of increased genetic structure in upstream areas. Overall, *E. lemniscatum* maintains gene flow across its range and is comprised of a single population. Given the small range and linear distribution of the species, we propose a stepping‐stone model of dispersal best explains the observed gene flow across its range, a phenomenon observed in other linearly distributed taxa (Gold, Burridge, & Turner, 2001; Pedersen, Ferchaud, Bertelsen, Bekkevold, & Hansen, 2017; Wagner & McCune, 2009). In general, our observation of higher‐than‐expected connectivity likely stems from adaptation of *E. lemniscatum* to temporally unstable and patchily distributed habitats.

### 4.1 Genetic diversity and conservation implications

Although direct comparison with studies of other species should be treated with caution due to differences in microsatellite markers used (Amos, Hutter, Schug, & Aquadro, 2003; Ellegren, 2004), *E. lemniscatum* exhibits low levels of allelic diversity (*N*_a_ and AR) like other imperiled darters, when compared to allelic diversity in nonimperiled darters (Table S3). Additionally, another indicator of reduced genetic diversity in *E. lemniscatum* was our microsatellite optimization results where we successfully amplified an additional 48 microsatellite primers, but 29 of those were monomorphic (the other 19 were difficult to accurately score). High allelic diversity at small numbers of neutral markers (such as the low number of markers typically used in microsatellite studies) is predictive of increased adaptive potential for quantitative traits (Vilas, Pérez‐Figueroa, Quesada, & Caballero, 2015), suggesting that the low allelic diversity seen in *E. lemniscatum* could be indicative of reduced adaptive potential compared to nonimperiled darters.

*Etheostoma lemniscatum* also exhibits evidence of recent bottleneck events. The extensive coal mining and logging that occurred in the Big South Fork watershed in the early twentieth century, and subsequent poor water quality and habitat conditions that led to habitat loss and degradation of spawning sites (O'Bara, Pennington, & Bonner, 1982; Rikard et al., 1986; USFWS, 2012), may have caused this recent drastic decline in population size. Anthropogenic effects on habitat are linked to bottleneck events and low genetic diversity in several other darter species, including the closely related *E. sitikuense*, which Moyer and Williams (2012) concluded was likely caused by contemporary anthropogenic impacts (coal mining, logging, etc.) instead of historical decreases in population size. The current range of *E. lemniscatum* (and that of the larger *Etheostoma percnurum* species complex, sensu Blanton & Jenkins, 2008) is considered relictual of a historically more widespread species. Population loss and range reduction were attributed to anthropogenic alterations to large river habitat (Blanton & Jenkins, 2008; Etnier & Starnes, 1993; Jenkins & Burkhead, 1994). This suggests both recent and historic bottlenecks may have contributed to the reduced genetic variation observed in *E. lemniscatum*, since bottlenecks can lead to increased genetic drift and inbreeding (Hedrick & Kalinowski, 2000; Nei, Maruyama, & Chakraborty, 1975; Spielman, Brook, & Frankham, 2004). However, unlike many other imperiled species that have experienced bottlenecks (Johnson et al., 2010; Noren, Godoy, Dalen, Meijer, & Angerbjorn, 2016; Taylor et al., 2017), *E. lemniscatum* showed no evidence of inbreeding and actually showed signatures of outbreeding in the area downstream of DJD. Brown et al. (2017) made a similar observation in highly endemic sulfide spring fishes, suggesting that inbreeding is not an inevitability of bottlenecked populations, especially in species, like *E. lemniscatum*, that have evolved in small geographic areas.

Eisenhour and Burr (2000) estimated a census size (*N*_c_) of 300–600 individuals, and a total of 200 and 100 individuals were estimated for the species in 2008 and 2009, respectively (Davis, 2010; Davis, Cook, & Smith, 2011). Our *N*_e_ estimate (*N*_e_ = 497, 95% CI = 315–1,060) and the total number of individuals observed in the course of our study (*N*_OBS_ = 271; Table S2) are slightly higher than these past census estimates. While these previous estimates could be inaccurate given the difficulty of sampling in a large river environment, it is possible that *E. lemniscatum* has undergone a population increase since the previous censuses. Since the species expanded into newly available habitats created when a portion of the river became free‐flowing due to construction on Wolf Creek Dam (which creates the Lake Cumberland reservoir), extending the known range of the species by approximately 8 km (USFWS, 2014), a population increase seems likely. Given the expansion of viable downstream habitats (although temporary) and observed improvements of habitat and water quality conditions throughout the Big South Fork (Worsham, Sundin, Nibblelink, Mengak, & Grossman, 2013), conditions may have been favorable for a recent population size increase, which could indicate the start of, or ongoing, recovery from past bottleneck events.

Given evidence of overall depressed genetic diversity, low *N*_e_, and past bottleneck events, *E. lemniscatum* likely has reduced evolutionary potential, and a diminished ability to weather stochastic events and changing environmental conditions (Frankham, 1995; Hoffmann, Sgrò, & Kristensen, 2017; Markert et al., 2010; Willi, Buskirk, & Hoffmann, 2006). For example, the 100/1,000 rule states that the minimum *N*_e_ necessary to maintain evolutionary potential in a species is 1,000 individuals (Frankham, Bradshaw, & Brook, 2014) indicating *E. lemniscatum* may lack sufficient *N*_e_ to maintain long‐term evolutionary potential. Given our genetic diversity results, the species small native range, the recent reinundation of the lower 8 km of the Big South Fork due to completion of repairs on Wolf Creek Dam, and continued sedimentation impacts stemming from various land uses (e.g., horse trails, logging, mining, and oil and gas exploration) (Olive & Marion, 2009; USFWS, 2012), we conclude that *E. lemniscatum* warrants continued federal protection. Because we found little genetic differentiation throughout our results, *E. lemniscatum* should be managed as a single unit. Since this species relies on dispersal between disjunct habitat patches to maintain gene flow across its range, anthropogenic in‐stream barriers (e.g., culverts, dams, etc.) or actions that reduce the permeability of intervening pool habitats, such as increased sedimentation, would negatively impact genetic exchange and diversity.

If *E. lemniscatum* is experiencing population growth and expansion as our data may indicate, the species should remain relatively stable as long as its habitat remains protected by the National Park Service and water quality keeps improving (Worsham et al., 2013). One threat to the species is horse trails, which increase sedimentation in the Big South Fork. Stabilizing and minimizing horse trails in riparian areas is an improvement that may benefit *E. lemniscatum*, and other sediment‐sensitive aquatic species, by reducing runoff and its negative effects on darter habitat (Olive & Marion, 2009). Additionally, the reinundation of the lower 8 km of its range may cause a population contraction and have negative genetic effects on the species overall.

### 4.2 Distribution of genetic variation in riverine systems

Unlike other riverine species that exhibit a downstream increase in intraspecific genetic diversity (DIGD) (Alp, Keller, Westram, & Robinson, 2012; Paz‐Vinas et al., 2015; Pilger et al., 2017), *E. lemniscatum* had fairly uniform amounts of genetic diversity across its range. However, our pairwise *F*_ST_ values and EEMS results indicate that *R1* (our most‐upstream reach) has reduced genetic input from across the rest of the species range. This is likely a signal of the expectation for increased genetic differentiation in upstream river sites, possibly due to more restricted gene flow in upstream directions from a downstream dispersal bias.

Heightened upstream genetic structure and DIGD is particularly pronounced in organisms inhabiting dendritic systems with many confluences and larger ranges (Crispo, Bentzen, Reznick, Kinnison, & Hendry, 2006; Ginson et al., 2015; Salisbury, McCracken, Keefe, Perry, & Ruzzante, 2016; Thomaz et al., 2016); however, linear river systems do not typically show strong signals of reduced upstream gene flow (Kanno et al., 2011; Paz‐Vinas et al., 2015; Thomaz et al., 2016). Thomaz et al. (2016) found a signal of DIGD in linear systems, but their models are based on a total range of 1,000 km, which suggests that a detectable signal of downstream‐biased dispersal (and the associated effects of DIGD and higher genetic differentiation in upstream areas) may not exist at smaller spatial scales such as that of *E. lemniscatum* (~38 km). In general, mainstem‐dwelling species (especially those with small ranges) display higher levels of connectivity due to the simplicity of dispersal in a mainstem linear habitat compared to dispersal in dendritic stream networks, which require longer and more complex movements between headwater areas to maintain population connectivity (Hitt & Angermeier, 2008; Hughes et al., 2009; Radinger & Wolter, 2014). Additionally, stronger signals of downstream‐biased dispersal exist in species that exhibit a passive dispersal phase (Pollux, Luteijn, Groenendael, & Ouborg, 2009), which *E. lemniscatum* does not. Ultimately, the simple linear distribution of *E. lemniscatum* in the Big South Fork mainstem and its small total range may prevent the formation of strong genetic signals from asymmetric downstream dispersal.

### 4.3 The effect of pools, distance, and rapids on gene flow in *Etheostoma lemniscatum*

Recovery of *E. lemniscatum* as a single genetic cluster was unexpected. We predicted that several variables, such as intervening pool habitats and distance, would exceed the dispersal capacity of *E. lemniscatum* and restrict gene flow. Furthermore, previous studies failed to detect *E. lemniscatum* in pools and noted that those reaches lack habitat typical of the species (Davis & Cook, 2010; Eisenhour & Burr, 2000). Our spatial autocorrelation analysis showed that pools did not restrict gene flow between adjacent localities unless they exceed the genetic patch size of 5.8 km, the distance at which gene flow becomes limited. Since most pools separating adjacent localities are 1–3 km long (Table S4), this finding suggests dispersal (and gene flow) occurs readily across most pools. This is further supported by our detection of significant positive spatial autocorrelation up to 2.45 km; therefore, localities separated by distances less than 2.45 km exhibit substantial dispersal and gene flow, even when those distances include pools.

Overall our IBD results indicate that distance is not a strong filter to gene flow. However, the observed genetic patch size in *E. lemniscatum* implies that long‐distance dispersal (e.g., small numbers of individuals moving from the downstream‐most to upstream‐most reaches) is not a common occurrence. Additionally, most darters are not known to make large seasonal long‐distance migrations (Page, 1983). Thus, neither long‐distance dispersal nor migration likely explains the maintenance of gene flow across the species range. Instead, we propose that gene flow persists across the range of *E. lemniscatum* via a stepping‐stone model and occurs indirectly over several generations. Species that have a stepping‐stone model of dispersal and gene flow often show a significant effect of geographic distance on genetic distance (Kimura & Weiss, 1964; Pedersen et al., 2017; Weston et al., 2016), similar to findings from our IBD and spatial autocorrelation analyses. Under this model, individuals from the most‐upstream and most‐downstream localities would not directly interbreed within the same generation. Instead, small numbers of effective dispersers over several generations are enough to prevent genetic structure from forming due to genetic drift (Mills & Allendorf, 1996; Spieth, 1974; Wright, 1931). At small spatial scales, like our study, even weak‐to‐moderate levels of gene flow, such as between adjacent habitat patches, are enough to prevent strong signatures of IBD (Menger et al., 2017; Phillipsen et al., 2015). Additionally, this level of dispersal between adjacent localities should readily occur since its linear distribution corresponds with a one‐dimensional stepping‐stone model, where dispersal is less complex than other habitat arrangements (Kimura & Weiss, 1964).

Both our Bayesian analyses and pairwise *F*_ST_ tests of individuals upstream and downstream of DJD recovered only low levels of genetic structure across this feature, indicating that the combination of an 11.7 km disjunction and presence of rapids acts as a filter, but not a barrier, to gene flow. *Etheostoma lemniscatum* also displays private alleles unique to the upstream or downstream areas of DJD, indicative of some degree of genetic drift. Conversely, our EEMS analysis suggested that DJD is a functional corridor for gene flow. Given that gene flow is restricted beyond 5.8 km in *E. lemniscatum*, we might expect DJD to create more genetic differentiation than observed. However, DJD is not completely devoid of possible habitat for *E. lemniscatum*: Eisenhour and Burr (2000) documented individuals at the mouth of Bear Creek, near the mid‐point of DJD. While *E. lemniscatum* is not regularly documented at this locality, it could still act as a stepping‐stone habitat patch that individuals may use temporarily to disperse across DJD. Because *E. lemniscatum* is small, individuals can presumably move through the reduced flows found in the benthic boundary layer in lotic environments (Carlson & Lauder, 2011). This would allow fish to avoid the fastest currents of rapids and disperse through the short rapid complexes found in DJD. Also, while our genetic patch size indicates that most movements occur within 5.8 km, movements beyond this distance are possible but likely rare.

### 4.4 Low genetic structure in a habitat specialist, dispersal‐limited species

Many darters are regarded as habitat specialists and dispersal‐limited due to their small body size and reduced or absent swim bladders (Knouft & Page, 2003; Radinger & Wolter, 2014). *Etheostoma lemniscatum* showed only low levels of genetic structure compared to that typically observed in habitat specialist, dispersal‐limited species (Fluker et al., 2014; Phillipsen et al., 2015; Wagner & McCune, 2009). Higher‐than‐expected dispersal and gene flow have been observed for other darters (Fluker et al., 2014; Ginson et al., 2015); for example, *Percina rex* showed evidence of some individuals dispersing up to 55 km and genetic panmixia up to 80 km (Roberts et al., 2013; Roberts, Angermeier, & Hallerman, 2016). Together, these studies suggest darters may not be as dispersal‐limited as once presumed, and that intrinsic biological features of organisms are not always predictive of dispersal capacity and gene flow.

There are several reasons why habitat specialists such as *E. lemniscatum* often contradict the assumptions of limited dispersal and higher genetic structure. For populations to remain stable in patchy habitats, it is expected that some level of effective dispersal is selected for to minimize isolation, inbreeding, and elevated extinction risk, as well as to allow individuals to colonize unoccupied habitat patches (Aars et al., 2006; Hanski & Gilpin, 1991; Saccheri et al., 1998). Additionally, dispersal may be particularly adaptive for organisms that occupy temporally variable habitats. When habitats shift or are lost, species that have adapted to this environment often display an ability to track that shifting habitat or move to new habitats if their current patch is lost (Denno, Roderick, Olmstead, & Dobel, 1991; Pereoglou et al., 2013; Wiens, 1976). Habitat of *E. lemniscatum* is likely temporally unstable, as the Big South Fork is in a gorge and experiences fast and drastic water‐level changes, leading to periods of high flow that can shift or eliminate *E. lemniscatum* habitat over time. Loss of riffle‐associated habitat can increase movements in other darters (Roberts & Angermeier, 2007), so movements of *E. lemniscatum* from shifting habitats (likely juveniles or adults since larvae become benthic immediately upon hatching; Douglas et al., 2013; Wallus & Simon, 2005) could account for the relatively high degree of population connectivity we observed. Thus, we propose that the maintenance of gene flow and evidence of dispersal across the range of *E. lemniscatum* reflects adaptation to a big river environment with patchy and, possibly, temporally unstable habitats.

When compared to other members of the *E. percnurum* species complex, *E. lemniscatum* has a more robust body and larger maximum size and also lives in the largest riverine habitat (Blanton & Jenkins, 2008; Eisenhour & Burr, 2000). Since fish species with larger maximum sizes exhibit longer movement distances (Radinger & Wolter, 2014), a comparatively larger, more robust body could reflect slight morphological adaptations to living and moving in a big river environment. Furthermore, while high reproductive investment (which *E. lemniscatum* exhibits) can indicate low dispersal ability and gene flow (Turner & Trexler, 1998), it can also contribute to higher recolonization success (Ensign, Leftwich, Angermeier, & Dolloff, 1997). This could allow *E. lemniscatum* to more readily populate new habitat patches as they become available in its temporally variable environment and explain how the species was able to relatively quickly recolonize habitat that became free‐flowing during the lowered reservoir levels on Lake Cumberland.

Habitat specialists may exhibit relaxed habitat requirements when dispersing (Fisher, Lambin, & Yletyinen, 2009; Palomares et al., 2000). This “generalist dispersal” strategy allows habitat specialists in patchy and temporally variable habitats to maintain genetic connectivity (Centeno‐Cuadros et al., 2011; Gauffre, Estoup, Bretagnolle, & Cosson, 2008; Pereoglou et al., 2013). In the case of *E. lemniscatum*, favorable or near‐favorable habitat patches may be more continuous through its range than we currently realize, which could aid generalist dispersal. Additionally, few surveys for *E. lemniscatum* have searched the deep, intervening pool habitats, and the species may occupy these deep pools more than currently acknowledged. There is precedent for this among darters: *Percina pantherina* is found in pools up to 5 m deep (Schaefer, Marsh‐Matthews, Spooner, Gido, & Matthews, 2003), but was previously only documented at depths less than 1 m (James & Maughan, 1989; Jones, Orth, & Maughan, 1984). The deepest *E. lemniscatum* has been documented is 1.2 m (Davis & Cook, 2010), but may occupy deeper waters where sampling for benthic species is difficult.

## 5 CONCLUSIONS

We found that a habitat specialist with presumed low dispersal ability displays a remarkably low level of genetic structure. The minor limits to gene flow observed were primarily attributable to distance, and possibly rapids, rather than intervening pool habitats. *Etheostoma lemniscatum* seems to have evolved dispersal adaptations in response to the patchy and, presumably, temporally variable habitat in its big river environment. Also, unlike many other riverine organisms, *E. lemniscatum* does not show strong signals of the expected distribution of genetic diversity that occurs due to asymmetric downstream dispersal. Its small range and linear distribution have likely contributed to the lack of these strong signals and the minimal levels of genetic differentiation observed. Our study and others (Ginson et al., 2015; Klauke et al., 2016; Saarinen, Reilly, & Austin, 2016; Weston et al., 2016) provide evidence that predictions of gene flow based on intrinsic biological characteristics of a species, particularly habitat specialization and dispersal ability, may not always hold true and responses may largely be species‐specific.

## CONFLICT OF INTEREST

None declared.

## AUTHOR CONTRIBUTIONS

R.E.B. and M.F.C. designed the study. B.A.W. performed molecular work and data analysis with help from R.E.B. and M.F.C. The manuscript was written by B.A.W. All authors participated in fieldwork and edited the manuscript.

## Data Availability

A spreadsheet containing all microsatellite genotypes for all sampled individuals is available on Dryad: https://doi.org/10.5061/dryad.xwdbrv19g. The spreadsheet includes each sampled individual's identification name and allele calls for all 20 loci, and the individual's corresponding locality and reach numbers.
