# Supplementary material for: Small fish, large river: Surprisingly minimal genetic structure in a dispersal‐limited, habitat specialist fish
Source: Ecol Evol. 2020 Feb 6;10(4):2253–68. doi: 10.1002/ece3.6064 (PMC7042738; doi:10.1002/ece3.6064)
Supplement: Supplementary file 1 [file ECE3-10-2253-s001.docx]

**Appendix 1**

**Table S1.** Primer sequences, PCR conditions and genotypic data from 20 microsatellite loci in *Etheostoma lemniscatum*. Genotypic data are summarized from our full data set of 163 individuals. N_a_ is number of alleles, and T_a_ is annealing temperature.

| **Primer** | **Primer sequence (5'-3')** | **Repeat  Motif** | **N_a_** | **Allelic  Range  (bp)** | **T_a_**  **(°C)** | |
| --- | --- | --- | --- | --- | --- | --- |
| **Elem035** | F: GGTGCCTTATGAAGAGCCTC R: GGCATACCCTACCCTAACCC | ACAT | 3 | 302-310 | 63⁰ | |
| **Elem011** | F: TGGGCAACTCATGTACAAGC R: ATTCCTCGCCACAGTCAGAC | AGAT | 9 | 345-385 | 60⁰ | |
| **Elem014** | F: TTCAACCAGATCAGCCCATC R: TGCAGCCTTTCCACAAACTC | AGAT | 6 | 301-329 | 60⁰ | |
| **Elem024** | F: TTGGGAGTGAGCCAAGACC R: GGTCGGAAAGAGGCACAAAC | AGAT | 5 | 360-376 | 63⁰ | |
| **Elem029** | F: AAAGAGATGGATAGCGTCAGAC R: GGACAATCACCCACTGTCTC | ACTC | 2 | 201-205 | 63⁰ | |
| **Elem016** | F: TGAAAGCAGACTAACGGGAG R: AACAGTTTGCCCAGCTTTGC | ACAG | 8 | 192-240 | 61⁰ | |
| **Elem028** | F: TTGCGTGCCAGAGTATTTGC R: TCGGTGGTGCTTTCAATGAG | AGAT | 6 | 369-393 | 60⁰ | |
| **Elem019** | F: CGTCTGTAGGTCATTTGGTGC R: CACATGCACAGGACCAGTAC | AGAT | 7 | 429-485 | 62⁰ | |
| **Elem045** | F: CCAGCCTGTGTGTGTTTCTAAC R: AGCTAAGTCTCTAGTGGCCG | AGAT | 5 | 244-264 | 63⁰ | |
| **Elem013** | F: GCCGAGGTAGAACTGGTTTG R: AGCAGGTGGGAGTATGAAGC | ACAT | 7 | 228-256 | 60⁰ | |
| **Elem018** | F: TAACACAAAGGCAGGGAAGC R: AAGGTTTGCAACAACATGAGG | AGAT | 7 | 418-442 | 60⁰ | |
| **Elem027** | F: CAACTGGACACTTCTGCCAC R: GCTCCAAGACCTGTAACCATG | ACAT | 6 | 277-297 | 60⁰ | |
| **Elem025** | F: TACTTTGGTCTGAATCGGCG R: CTCTTGGGTTCACAATGCCC | AGAT | 3 | 227-251 | 61⁰ | |
| **Elem004** | F: ATTGCGTCCTTTGCCTGTTC R: TCAGCAGTGATACATGGGTG | AGAT | 12 | 243-311 | 63⁰ | |
| **Elem032** | F: TGTGCTCAGTCACACATCCC R: GGAAGAGAGATGCAAGGAAGC | ACAG | 4 | 197-213 | 63⁰ | |
| **Elem010** | F: TGGACTTGAAACAGACTGTGG R: AGGTAGATGTGGGTGGGTAG | AGAT | 8 | 250-294 | 65⁰ | |
| **Elem085** | F: CTTGCTACTGGATGCCTTCG R: AGGGTTGGTTAGGATGGATGG | AGAT | 5 | 171-187 | 65⁰ | |
| **Elem089** | F: TGTCTACACAGGTCACACAGG R: GCAAATGGACAGATGGACGG | AGAT | 7 | 236-268 | 65⁰ | |
| **Elem092** | F: ACACATTGACACAAACCCGG R: GGGACCGGAGCTATACATCC | AGAT | 7 | 348-392 | 65⁰ |  |
| **Elem093** | F: CCACTGTCAAAGAACTAGCCC R: GCAGAGAGGAGATGACCCTG | AGAT | 3 | 430-446 | 65⁰ |  |

**Table** **S2.** Locality information and number of individuals of *Etheostoma lemniscatum* captured (N_CAPT_) and observed (N_OBS_) at each of the 18 sites examined. All captured individuals were used in genetic analyses. Locality numbers correspond to those used in Figure 2 and in Table S4.

| **Locality Number** | **Location** | **Date Collected** | **N_CAPT_** | **N_OBS_** |
| --- | --- | --- | --- | --- |
| 1 | Mill Creek | 23-Oct-15 | 16 | 18 |
| 2 | Station Camp Creek | 4-Aug-15 | 20 | 20 |
| 3 | Downstream of Parch Corn Creek | 5-Aug-15 | 5 | 6 |
| 4 | Between Big Branch & Cold Spring | 4-Sep-15 | 17 | 32 |
| 5 | Big Island | 5-Sep-15 | 17 | 31 |
| 6 | Williams Creek | 5-Sep-15 | 2 | 10 |
| 7 | Upstream of Hurricane Creek | 5-Sep-15 | 9 | 16 |
| 8 | Difficulty Creek | 5-Sep-15 | 2 | 4 |
| 9 | Between Cub Branch and Troublesome Creek | 6-Sep-15 | 13 | 20 |
| 10 | Upstream of Oil Well Branch | 6-Sep-15 | 16 | 27 |
| 11 | Upstream Devils Creek, Blue Heron | 16-Sep-15 | 5 | 7 |
| 12 | Between Devils Creek and Roaring Paunch Creek | 14-Sep-15 | 2 | 12 |
| 13 | Mouth of Roaring Paunch Creek | 22-Sep-15 | 6 | 16 |
| 14 | Downstream 1.3km Roaring Paunch Creek | 15-Sep-15 | 14 | 20 |
| 15 | Upstream 400m Stover Branch | 15-Sep-15 | 4 | 6 |
| 16 | Downstream of Rock Creek | 21-Sep-15 | 5 | 14 |
| 17 | Downstream of KY HWY 92 Crossing | 17-Sep-15 | 9 | 11 |
| 18 | Mouth of Lick Creek | 23-Sep-15 | 1 | 1 |
|  |  | **Total** | 163 | 271 |
|  |  | **Mean** | 9 | 15 |

**Table S3**. Genetic diversity metrics summarized from other studies of imperiled and non-imperiled darter species as compared to *Etheostoma lemniscatum*. Species are grouped by conservation status. Metrics included are mean alleles per locus (N_a_), allelic richness (AR), observed (H_O_) and expected (H_E_) heterozygosity. A dash indicates that genetic diversity metric was not reported.

| **Species** | **N_a_** | **AR** | **H_O_** | **H_E_** | **Citation** |
| --- | --- | --- | --- | --- | --- |
| **Federally Endangered** |  |  |  |  |  |
| Tuxedo Darter (*Etheostoma lemniscatum*) | 6.0 | 4.75 - 4.91 | 0.601 | 0.597 |  |
| Citico Darter (*Etheostoma sitikuense*) | - | 2.67 | 0.246 | 0.228 | Moyer and Williams 2012 |
| Watercress Darter (*Etheostoma nuchale*) | 5.6 | 4.41 - 6.28 | 0.671 | 0.676 | Fluker et al. 2010 |
| Roanoke Logperch (*Percina rex*) | - | 2.70 - 9.70 | 0.699 | 0.719 | Roberts et al. 2013 |
| Bluemask Darter (*Etheostoma akatulo*) | 3.4 | 2.16 - 4.08 | 0.292 | 0.275 | Robinson et al. 2013 |
| Fountain Darter (*Etheostoma fonticola*) | - | 6.99 - 8.61 | 0.625 | 0.641 | Olsen et al. 2016 |
| **Federally Threatened** |  |  |  |  |  |
| Okaloosa Darter (*Etheostoma okaloosa*) | 24.0 | - | 0.599 | 0.661 | Austin et al. 2011 |
| Slackwater Darter (*Etheostoma boschungi*) | 6.74 | 3.34 - 9.38 | 0.650 | 0.670 | Fluker et al. 2014a |
| Kentucky Arrow Darter (*Etheostoma sagitta spilotum*) | 3.70 | - | 0.426 | 0.425 | Blanton et al. 2019 |
| **Imperiled** |  |  |  |  |  |
| Yazoo Darter (*Etheostoma raneyi*) | - | 4.66 - 6.89 | 0.749 | 0.687 | Sterling et al. 2012 |
| Arkansas Darter (*Etheostoma cragini*) | - | 2.50 - 5.30 | 0.409 | 0.447 | Fitzpatrick et al. 2014 |
| Tuscumbia Darter (*Etheostoma tuscumbia*) | 5.53 | 1.74 - 7.50 | 0.570 | 0.570 | Fluker et al. 2014a |
| **Non-Imperiled** |  |  |  |  |  |
| Greenside Darter (*Etheostoma blennioides*) | 9.31 | - | 0.654 | 0.686 | Beneteau et al. 2008 |
| Gulf Darter (*Etheostoma swaini*) | 12.41 | 9.87 - 12.44 | 0.797 | 0.870 | Fluker et al. 2010 |
| Rainbow Darter (*Etheostoma caeruleum*) | 10.69 | 6.33 - 7.34 | 0.857 | 0.842 | Davis et al. 2014 |
| Tallapoosa Darter (*Etheostoma tallapoosae*) | 9.98 | 6.59 - 12.99 | 0.688 | 0.689 | Fluker et al. 2014b |
| Eastern Sand Darter (*Ammocrypta pellucida*) | - | 2.64 - 5.87 | 0.673 | 0.692 | Ginson et al. 2015 |

**Table S4.** Distances between adjacent *Etheostoma lemniscatum* localities shown in Figure 2 and Table S2. DJD identifies the reach encompassing Devil’s Jump Disjunction. Pairwise geographic distances between all localities used in our IBD analysis provided in Table 2.

| **Locality Number** | **Distance from Previous Site** |
| --- | --- |
| 1 | - |
| 2 | 2.65 km |
| 3 | 1.66 km |
| 4 | 2.39 km |
| 5 | 1.30 km |
| 6 | 3.17 km |
| 7 | 1.32 km |
| 8 | 1.42 km |
| 9 | 1.87 km |
| 10 | 2.65 km |
| 11 | 11.74 km (DJD) |
| 12 | 0.65 km |
| 13 | 0.50 km |
| 14 | 1.30 km |
| 15 | 1.27 km |
| 16 | 2.28 km |
| 17 | 0.95 km |
| 18 | 1.49 km |

**
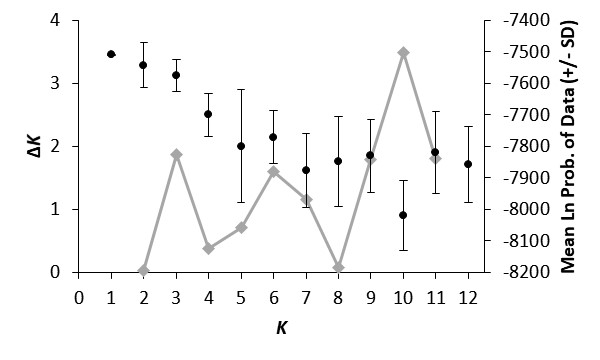
**

**(a)**

17

15

12


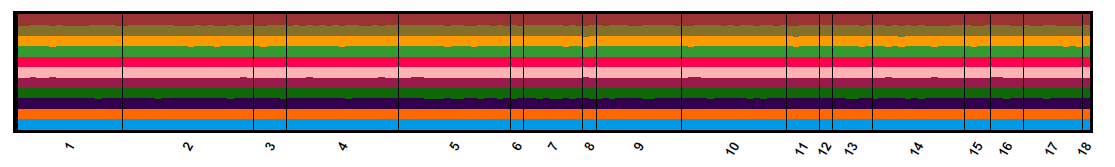


1

2

3

4

5

6

7

8

9

10

11

13

14

16

18

**(b)**


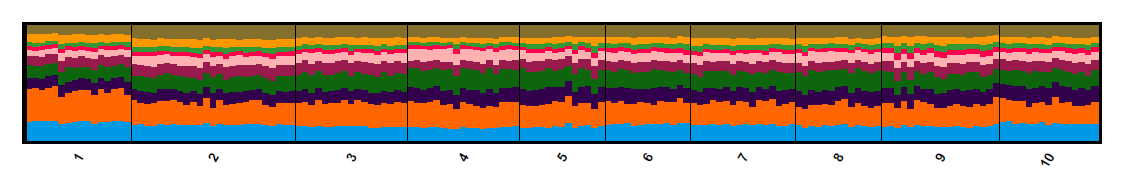


*R1*

*R2*

*R3*

*R4*

*R5*

*R6*

*R7*

*R8*

*R9*

*R10*

**(c)**

**Figure S1**. (a) Estimates for the most likely number of genetic clusters (*K*) based on analysis of 20 microsatellite loci and 163 individuals of *Etheostoma lemniscatum* in STRUCTURE with the 10 stream reaches (*R1-R10*) designated in Figure 2 as priors using the LOCPRIOR function. Black circles with standard deviation error bars denote the mean log likelihood over 5 iterations for each *K* and correspond with the right y-axis values, and gray diamonds represent Δ*K* values for each *K* and correspond with the left y-axis. Values of *K* examined were the total number of reaches + 2. (b) STRUCTURE plot for the best supported *K*, *K* = 11, for the initial structure run with no a priori population assumptions. (c) STRUCTURE plot for the best supported *K*, *K* = 10, for the subsequent STRUCTURE run with LOCPRIOR included with our 10 reach groupings used as the prior.

**(a)**


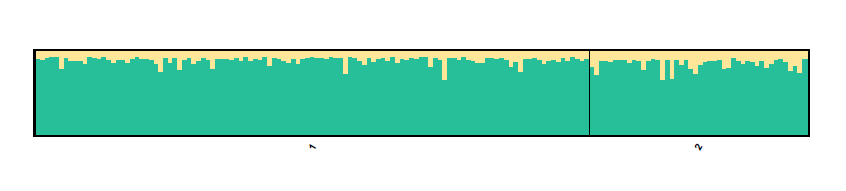


Upstream DJD

Downstream DJD

**DJD**

**(b)**

**
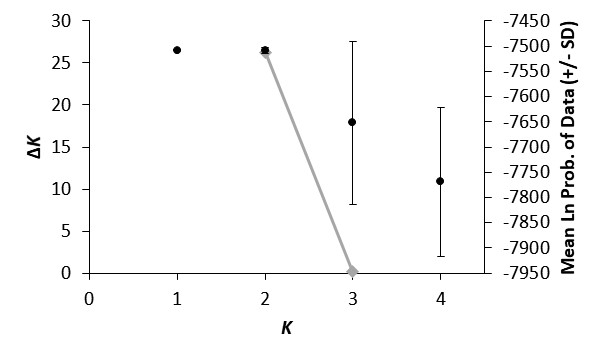
Figure S2.** Estimate for the most likely number of genetic clusters (*K*) based on analysis of 20 microsatellite loci and 163 individuals of *Etheostoma lemniscatum* in STRUCTURE. (a) LOCPRIOR model with the areas upstream (*R1-R7*) and downstream (*R8-R10*) of DJD used as the prior. Black circles with standard deviation error bars denote the mean log likelihood over 5 iterations for each *K* and correspond with the right y-axis values, and gray diamonds represent Δ*K* values for each *K* and correspond with the left y-axis. (b) STRUCTURE plot averaged across all five iterations for the best supported *K* according to the Δ*K* method, *K* = 2. Each vertical bar represents an individual’s genotype and colors of each bar indicate the proportion of the genotype assigned to the area upstream or downstream of DJD.

**
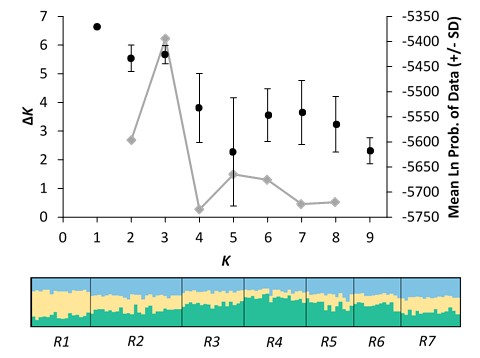
**

**(a)**


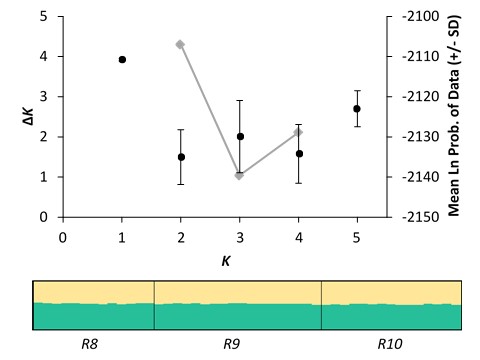


**(b)**

**Figure S3.** Estimate for the most likely number of genetic clusters (*K*) based on analysis of 20 microsatellite loci and 163 individuals of *Etheostoma lemniscatum* in STRUCTURE. Black circles with standard deviation error bars denote the mean log likelihood over 5 iterations for each *K* and correspond with the right y-axis values, and gray diamonds represent Δ*K* values for each *K* and correspond with the left y-axis. STRUCTURE plots averaged across all five iterations for the best supported *K* according to the Δ*K* method. Each vertical bar represents an individual’s genotype and colors of each bar indicate the proportion of the genotype assigned to a proposed cluster. (a) LOCPRIOR model for the area upstream of DJD with *R1-R7* used as a prior and STRUCTURE plot for best supported *K* using the Δ*K* method, *K* = 3 (b) LOCPRIOR model for the area downstream of DJD with *R8-R10* used as a prior and STRUCTURE plot for best supported *K* using the Δ*K* method, *K* = 2.

**References**

Austin, J. D., H. L. Jelks, B. Tate, A. R. Johnson, and F. Jordan. 2011. Population genetic structure and conservation genetics of threatened Okaloosa darters (*Etheostoma okaloosae*). Conservation Genetics 12:981–989.

Beneteau, C. L., N. E. Mandrak, and D. D. Heath. 2008. The effects of river barriers and range expansion of the population genetic structure and stability in Greenside Darter (*Etheostoma blennioides*) populations. Conservation Genetics 10:477–487.

Blanton, R. E., M. F. Cashner, M. R. Thomas, S. L. Brandt, and M. A. Floyd. 2019. Increased habitat fragmentation leads to isolation among and low genetic diversity within populations of the imperiled Kentucky Arrow Darter (*Etheostoma sagitta spilotum*). Conservation Genetics:1–14.

Davis, D. J., A. C. Wieman, and P. B. Berendzen. 2014. The influence of historical and contemporary landscape variables on the spatial genetic structure of the rainbow darter (*Etheostoma caeruleum*) in tributaries of the upper Mississippi River. Conservation Genetics 16:167–179.

Fitzpatrick, S. W., H. Crockett, and W. C. Funk. 2014. Water availability strongly impacts population genetic patterns of an imperiled Great Plains endemic fish. Conservation Genetics 15:771–788.

Fluker, B. L., B. R. Kuhajda, and P. M. Harris. 2014a. The influence of life-history strategy on genetic differentiation and lineage divergence in darters (Percidae: Etheostomatinae). Evolution 68:3199–3216.

Fluker, B. L., B. R. Kuhajda, and P. M. Harris. 2014b. The effects of riverine impoundment on genetic structure and gene flow in two stream fishes in the Mobile River basin. Freshwater Biology 59:526–543.

Fluker, B. L., B. R. Kuhajda, N. J. Lang, and P. M. Harris. 2010. Low genetic diversity and small long-term population sizes in the spring endemic watercress darter, *Etheostoma nuchale*. Conservation Genetics 11:2267–2279.

Ginson, R., R. P. Walter, N. E. Mandrak, C. L. Beneteau, and D. D. Heath. 2015. Hierarchical analysis of genetic structure in the habitat-specialist Eastern Sand Darter (*Ammocrypta pellucida*). Ecology and Evolution 5:695–708.

Moyer, G. R., and A. S. Williams. 2012. Genetic assessment of Abrams Creek reintroduction program for the federally threatened yellowfin madtom (*Noturus flavipinnis*), and endangered smoky madtom (*Noturus baileyi*) and Citico darter (*Etheostoma sitikuense*).

Olsen, J. B., A. P. Kinziger, J. K. Wenburg, C. J. Lewis, C. T. Phillips, and K. G. Ostrand. 2016. Genetic diversity and divergence in the fountain darter (*Etheostoma fonticola*): implications for conservation of an endangered species. Conservation Genetics 17:1393–1404.

Roberts, J. H., P. L. Angermeier, and E. M. Hallerman. 2013. Distance, dams and drift: what structures populations of an endangered, benthic stream fish? Freshwater Biology 58:2050–2064.

Robinson, J. D., J. W. Simmons, A. S. Williams, and G. R. Moyer. 2013. Population structure and genetic diversity in the endangered bluemask darter (*Etheostoma akatulo*). Conservation Genetics 14:79–92.

Sterling, K. A., D. H. Reed, B. P. Noonan, and M. L. Warren Jr. 2012. Genetic effects of habitat fragmentation and population isolation on *Etheostoma raneyi* (Percidae). Conservation Genetics 13:859–872.
